# Analyzing and predicting patient admissions related to acute heat at the Chemnitz Hospital (Germany)

**DOI:** 10.1186/s13690-025-01789-9

**Published:** 2025-11-26

**Authors:** J. Thiel, E. Henke, M. Sedlmayr, A. A. Baumann, J. Weidner, A. Staudt

**Affiliations:** 1 Institute for Medical Informatics and Biometry, Faculty of Medicine and University Hospital Carl Gustav Carus, TUD Dresden University of Technology, Fetscherstraße 74, 01307 Dresden, Germany; 2 Department of Systems Biology and Bioinformatics, University of Rostock, Rostock, Germany; 3 Institute and Policlinic of Occupational and Social Medicine, Faculty of Medicine and University Hospital Carl Gustav Carus, TUD Dresden University of Technology, Fetscherstraße 74, 01307 Dresden, Germany

**Keywords:** Heat impact on health, Heat-related hospitalization, Heatwaves, Climate change, Forecast, Prediction

## Abstract

**Background:**

Climate change is leading to an increase in acute heatwaves, thereby raising risks to human health and healthcare systems. The aim of this study was to quantify the relationship between heat and hospital admissions, and to develop a predictive model for patient admissions.

**Methods:**

A retrospective secondary data analysis was conducted for the period 2018–2023, using data from Chemnitz Hospital and the Regional Climate Information Service of TU Dresden. Hospital admissions with the following diagnoses were analyzed: E86 (Volume depletion), I20 (Angina pectoris), I21 (Acute myocardial infarction), I63 (Cerebral infarction), I64 (Stroke, unspecified as hemorrhagic or ischemic), J44 (COPD), L55 (Sunburn), T67 (Effects of heat and light). Meteorological variables considered included temperature, humidity, air pressure, and daily sunshine hours. Analyses included correlations between daily maximum temperatures (≥ 23°C and ≥ 30°C) and hospital admissions, as well as linear and non-linear regression analyses., both univariate (maximum temperature) and multivariate (humidity, air pressure, sunshine hours).

**Results:**

A positive correlation between maximum temperature and hospital admissions was observed: *r* = 0.44 (*p* > 0.05) for ≥ 23°C and *r* = 0.88 (*p* < 0.01) for ≥ 30°C. Regression analyses suggested a non-linear relationship (U-shaped pattern with alternating curvature) between temperature and hospital admissions for days with a maximum temperature between 23°C and 29°C, and a linear effect for days above 30°C, additionally influenced by air pressure, humidity, and sunshine hours.

**Conclusion:**

The results indicate a complex association between heat and hospital admissions. Based on these findings, a predictive model was developed to forecast patient admissions. The study highlights the importance of primary preventive measures and the consideration of moderate heat stress in healthcare planning.

**Supplementary Information:**

The online version contains supplementary material available at 10.1186/s13690-025-01789-9.

**Table:** 

Text box 1. Contributions to the literature
• Investigates the relationship between heat exposure and hospital admissions in Eastern Germany
• Quantifies the health impacts of both moderate and extreme heat events
• Provides some of the first region-specific insights into heat-related health effects in Germany
• Among the first studies to explore predictive models for hospital resource planning during heat events in the German healthcare context

## Background

Global climate change is expected to drive further temperature increases, with both the intensity and duration of heatwaves projected to rise [1]. Its effects are far-reaching, manifesting in extreme weather events such as heatwaves, droughts, floods, and storms [2]. The World Health Organization (WHO) assumes that such events will continue to increase as a result of climate change and expects up to 250,000 deaths and an additional burden on the health care system between 2030 and 2050 [1]. An analysis of deaths in the summer of 2022, the hottest summer in Europe since weather records began, shows over 60,000 heat-related deaths. Most of these deaths were recorded in Italy, Spain, and Germany [3]. Meta-analyses show statistically significant increases in healthcare utilization during heat waves worldwide [4]. Risk groups include young children, older people, and people with pre-existing conditions or physically demanding jobs [5–7].

In Germany, rising temperatures and the resulting increase in morbidity represent a key challenge as a consequence of climate change [1]. This is particularly relevant because the optimal temperature for humans is around 17–19°C, and deviations from this can contribute to an increase in mortality and morbidity [3, 8]. Rising air temperatures and the associated health risks pose a significant public health challenge [1], as heat impacts human health in multiple ways, including dehydration, heat stroke [4], and an increased incidence of cardiovascular diseases such as heart attacks and heart failure [4]. Furthermore, respiratory conditions such as asthma and chronic obstructive pulmonary disease (COPD) are linked to high temperatures [9]. The growing burden of these illnesses places additional strain on healthcare systems [10]. However, it is not clearly defined which temperature limits are to be considered heat and when they are relevant to health risks. This is evident, for example, in the WHO recommendations for the prevention of heat-related illnesses, which use different thresholds between 25°C and 35°C depending on individual and other climatic factors [11]. The Robert-Koch-Institute, the institute responsible for managing health risks in Germany [12], uses a similar approach and uses fixed thresholds starting at around 20°C to investigate heat-related health effects depending on the region, age group, and observation period [13].

To mitigate these impacts, the German Federal Ministry for the Environment, Nature Conservation, Building, and Nuclear Safety published recommendations in 2017 for the development of heat action plans to protect public health, aiming for implementation at state and municipal levels [14, 15]. Other countries have already adopted nationwide strategies. In the United Kingdom, for example, heat warnings are issued based on climatic conditions, triggering targeted interventions such as public notifications via SMS or email and the provision of cooling rooms in hospitals [16]. In addition, approaches for local predictions of hospital occupancy during heatwaves are being developed, as this can help to identify and mitigate possible overloading of the healthcare system at an early stage. For example, Nishimura et al. utilized retrospective treatment and weather data to forecast patient numbers in response to heat in Nagoya, Japan [17]. The integration of digital tools can further enhance such predictive capabilities [18].

To advance digitalization in the German healthcare system, the Federal Ministry of Education and Research is supporting initiatives such as the Medical Informatics Initiative and six Digital Health Progress Hubs. One of these hubs, the Medical Informatics Hub in Saxony (MiHUBx), aims to develop a tool for regional forecasting of hospital occupancy during acute heat events [19]. The development of such a statistical model requires a precise understanding of the relationship between heat and hospital utilization at the regional level. To our knowledge, there is hardly any evidence available to inform such a prediction model. Existing analyses in Germany known to us concentrate on individual risk groups such as patients over 65 years of age [20], do not take sufficient account of relevant diseases (e.g. cardiovascular diseases) [5–7] or refer to entire federal states instead of individual hospitals or planning regions [21]. Therefore, this study aims to expand the evidence regarding the association between acute heat and hospital admissions, thus increasing statistical power through a larger patient cohort and enabling the development of a prediction model.

## Methods

### Data sources and data preparation

Clinical data were obtained from Chemnitz Hospital via its affiliated Data Integration Center (DIC) Chemnitz. Chemnitz Hospital is a maximum care hospital in the region of south-west Saxony in Germany [22]. All patients admitted as inpatients (including emergency department) during the study period from January 1, 2018, to December 1, 2023 were included.

The selection of analyzed diseases and other factors, such as sociodemographic and treatment-specific data, was based on a literature review [5–7], a mapping of heat-related illnesses (Thiel, Sedlmayr, et al., 2025), and was consistent with the analyses conducted with data from Dresden University Hospital [5–7]. Additionally, ethical approval for this study was obtained from the Ethics Committee of TU Dresden (SR-EK-479112023/2024).

The study included demographic data such as patient age (grouped in 5-year intervals: 0–4 years, 5–9 years, etc.), gender, and place of residence (based on the first three digits of the postal code). Further we collected data about visit start and end dates, and diagnosis codes related to the primary reason for hospitalization, classified according to the International Classification of Diseases, 10th Revision, German Modification (ICD-10-GM) [23].

For this study, the following ICD-10-GM codes were selected literature-based and retrieved from DIC Chemnitz:

- E86 (Volume depletion)
- I20 (Angina pectoris)
- I21 (Acute myocardial infarction)
- I63 (Cerebral infarction)
- I64 (Stroke, unspecified as hemorrhagic or ischemic)
- J44 (Chronic obstructive pulmonary disease)
- L55 (Sunburn)
- T67 (Effects of heat and light)

Additionally, all subordinate diagnosis codes within each diagnostic category were included. For example, the analysis of I20 also encompassed the subcodes I20.0, I20.1, I20.8, and I20.9. However, for the final analysis, subcategories were aggregated at the hierarchical group level, and all diagnostic codes within I20 were analyzed collectively to provide a broader overview of disease patterns.

Climate data were obtained from the Regional Climate Information System (ReKIS) of the Technical University of Dresden [24]. The analysis utilized daily mean temperature and daily maximum air temperature from a weather station in Chemnitz. In addition, the number of hours of sunshine, average humidity, and average air pressure for each day were included as control variables, as these parameters have been frequently reported in the literature [5–7].

Defining specific heat thresholds was necessary, as no universally accepted definition of heat nor standardized threshold values could be identified in the international literature. In Germany, the Robert Koch Institute, as the competent authority, uses various threshold values starting at 20 °C [13], while other studies in Germany have found correlations starting at 23 °C [5–7, 25]. Therefore, this study uses ≥ 23 °C as the threshold value for warm days. In addition, another threshold value was set at a daily maximum temperature of ≥ 30 °C as a heat day. This definition aligns with the commonly accepted definition of a heat day in Germany [26].

### Statistical analysis

The statistical analysis was conducted using RStudio (R Version 4.4.1) [27]. First, clinical data were analyzed by determining the total number of hospital admissions as well as the yearly case numbers. Additionally, the absolute frequency of primary diagnoses and the corresponding mean length of hospital stay were calculated. To assess sociodemographic characteristics, the gender distribution within the total study population was determined. Furthermore, age distribution was analyzed both in general and in relation to specific diagnoses and gender.

The analysis of climate data included calculating the absolute frequency of warm days and heat days for each year as well as determining the mean temperature. The mean temperature was computed for the entire study period and separately for each calendar month.

To assess the correlation between heat and hospital admissions, a lag of 0 days was applied, meaning that admissions were linked to the heat event on the same day. According to Peng et al., the strongest association is expected with a 0-day lag [28]. In our analysis, the effect was quantified using Pearson’s correlation coefficient for linear correlation, with statistical significance (t-test) assessed at a 5% significance level. Across all tables, significance levels are indicated as follows: * *p* < 0.05, ** *p* < 0.01, *** *p* < 0.001. In addition, Bayes Factors (BF) were calculated, assuming a normal distribution with an expected correlation of 0.4 (derived from a previous analysis [5–7]), that provide additional information about how sensitive the data are to distinguish null and alternative hypotheses. BF values below 0.33 were considered as evidence against, values above 3 as evidence for an association between acute heat and hospital admissions, and intermediate values as indicating inconclusive evidence, sometimes referred to as data insensitivity [29]. The average number of daily hospitalizations was determined to minimize potential biases due to uneven distribution of certain temperature values. Analyses for individual diagnoses were only carried out for diagnoses that accounted for at least 1% of the total cases in the data set. This threshold ensures sufficient sample size. Therefore, also the temperature values were rounded to whole numbers for all calculations.

In addition, regression analysis was performed for all diseases combined, the functional representation of all approaches for regression is shown in Appendix 1. First, a linear regression analysis was performed between daily maximum temperature and number of patient admissions. Subsequently, hours of sunshine, air temperature, and air pressure were included as control variables and a multiple linear regression was calculated. Furthermore, non-linear relationships were investigated using a generalized additive model (GAM). GAMs allow flexible, smooth functions of predictor variables to capture complex non-linear effects. In our analysis these were modeled using splines, with the effective degrees of freedom (edf) used to assess the linearity of each effect. An edf of 1.0 means perfect linearity. The further the edf deviates from 1.0, the less linear the relationships are [30]. For GAM, too, a univariate analysis was first performed between maximum temperature and patient admissions. Subsequently, hours of sunshine, air temperature, and air pressure were also included in the analyses as control variables. All diagnoses included in the study included diagnoses were taken into account for all regression analyses, and individual models were created for the thresholds of warm days (≥ 23 °C) and heat days (≥ 30°C). No stratification or separate analysis by patient age were conducted in this study.

## Results

### Description of the heat-related illnesses and the study population

**Fig. 1:**
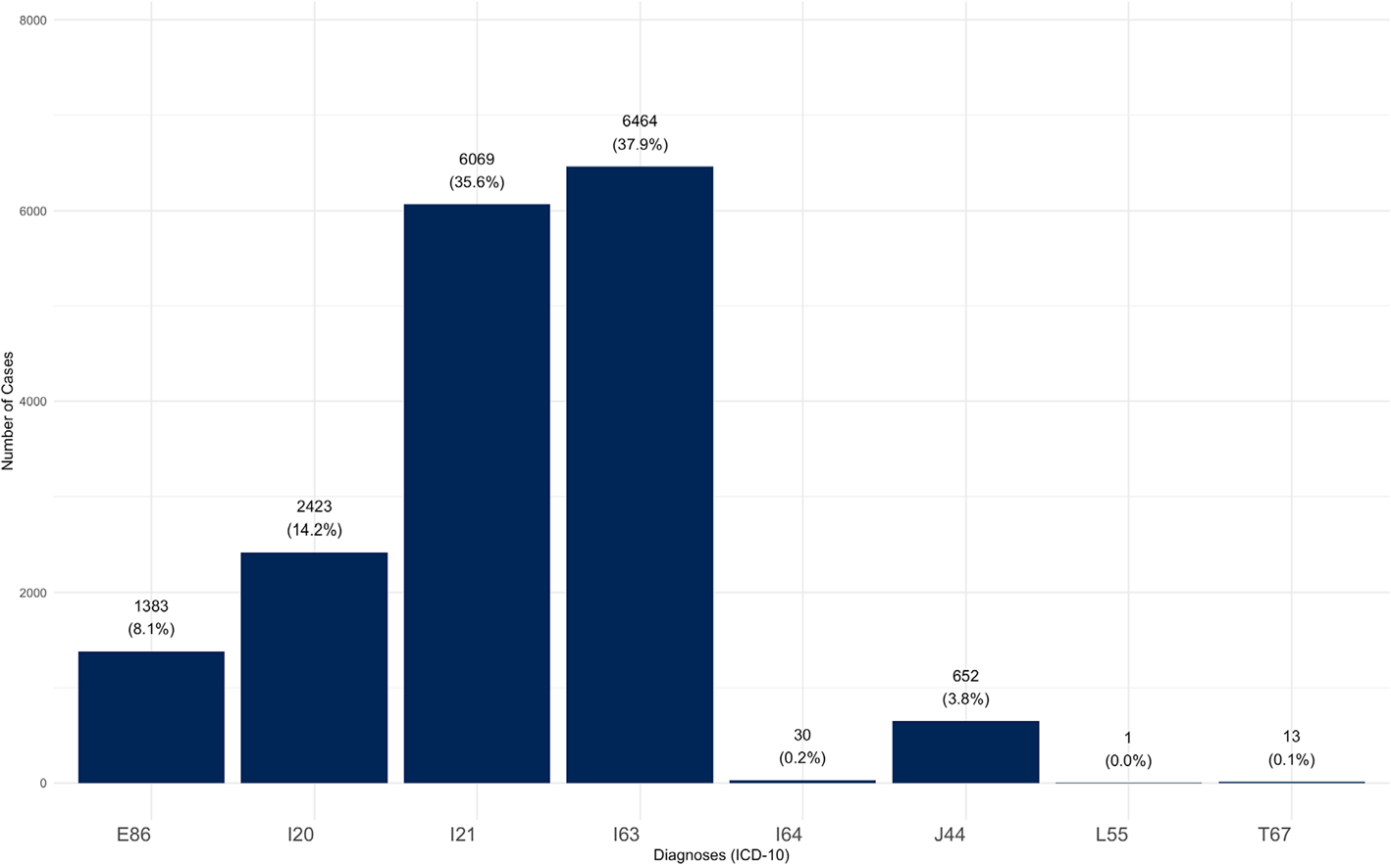
Frequency of the specified diagnoses in the observation period at Chemnitz Hospital (Germany), 2018–2023

In total, data on 15,011 patients with 17,035 treatment cases were provided by the DIC Chemnitz during the observation period. The annual case numbers ranged from 2,688 to 3,045 cases per year and cumulatively amounted to 17,035 over the entire study period (Table 1). With a relative frequency of 37.9%, the diagnosis I63 (Cerebral infarction) was the most frequent diagnosis, followed by the cardiovascular diseases I21 (35.6%) and I20 (14.2%). The diagnoses I64 (0.2%), T67 (0.1%) and L55 (0.1%) encompassed less than 1% of all treatment cases and were therefore not analyzed individually (Fig. 1). On average, the longest length of stay was found for diagnosis I63 (*Mean M* = 13.09 days; *Standard deviation SD* = 10.45 days) and the shortest length of stay for diagnosis I20 (*M* = 3.87 days; *SD* = 3.39 days). The lengths of stay are shown in Table 2.

**Table 1:** Number and percentage of treatment cases per year, in total and divided by analyzed and other (I64, T67, L55) diagnosis codes at Chemnitz Hospital (Germany), 2018–2023

**Year**	**Total Cases**	**E86 -Volume depletion**	**I20—Angina pectoris**	**I21—Acute myocardial infarction**	**I63—Cerebral infarction**	**J44—COPD**	**Other**
2018	2,841 (100%)	253 (8,9%)	353 (12,4%)	1,016 (35,8%)	1,082 (38,1%)	126 (4.4%)	11 (0.4%)
2019	3,045 (100%)	243 (8,0%)	455 (14,9%)	1,055 (34,6%)	1,150 (37,8%)	134 (4.4%)	8 (0.3%)
2020	2,821 (100%)	166 (5,9%)	510 (18,1%)	1,012 (35,9%)	1,057 (37,5%)	74 (2.6%)	2 (0.1%)
2021	2,759 (100%)	183 (6,6%)	419 (15,2%)	1,040 (37,7%)	1,030 (37,3%)	78 (2.8%)	9 (0.3%)
2022	2,688 (100%)	197 (7,3%)	370 (13,8%)	968 (36,0%)	1,037 (38,6%)	105 (3.9%)	11 (0.4%)
2023	2,881 (100%)	341(11,8%)	316 (11,0%)	978 (33,9%)	1,108 (38,5%)	135 (4.7%)	3 (0.1%)
Total	17,035	1,383	2,423	6,069	6,464	652	696

**Table 2:** Mean length of stay in days for patients by diagnosis at Chemnitz Hospital (Germany), 2018–2023

Diagnosis code	Mean (Standard deviation)
All diagnoses	9.00 (9.50)
E86—Volume depletion	7.25 (6.42)
I20—Angina pectoris	3.87 (3.39)
I21—Acute myocardial infarction	6.85 (8.94)
I63—Cerebral infarction	13.09 (10.45)
J44—COPD	11.72 (8.90)

**Fig. 2:**
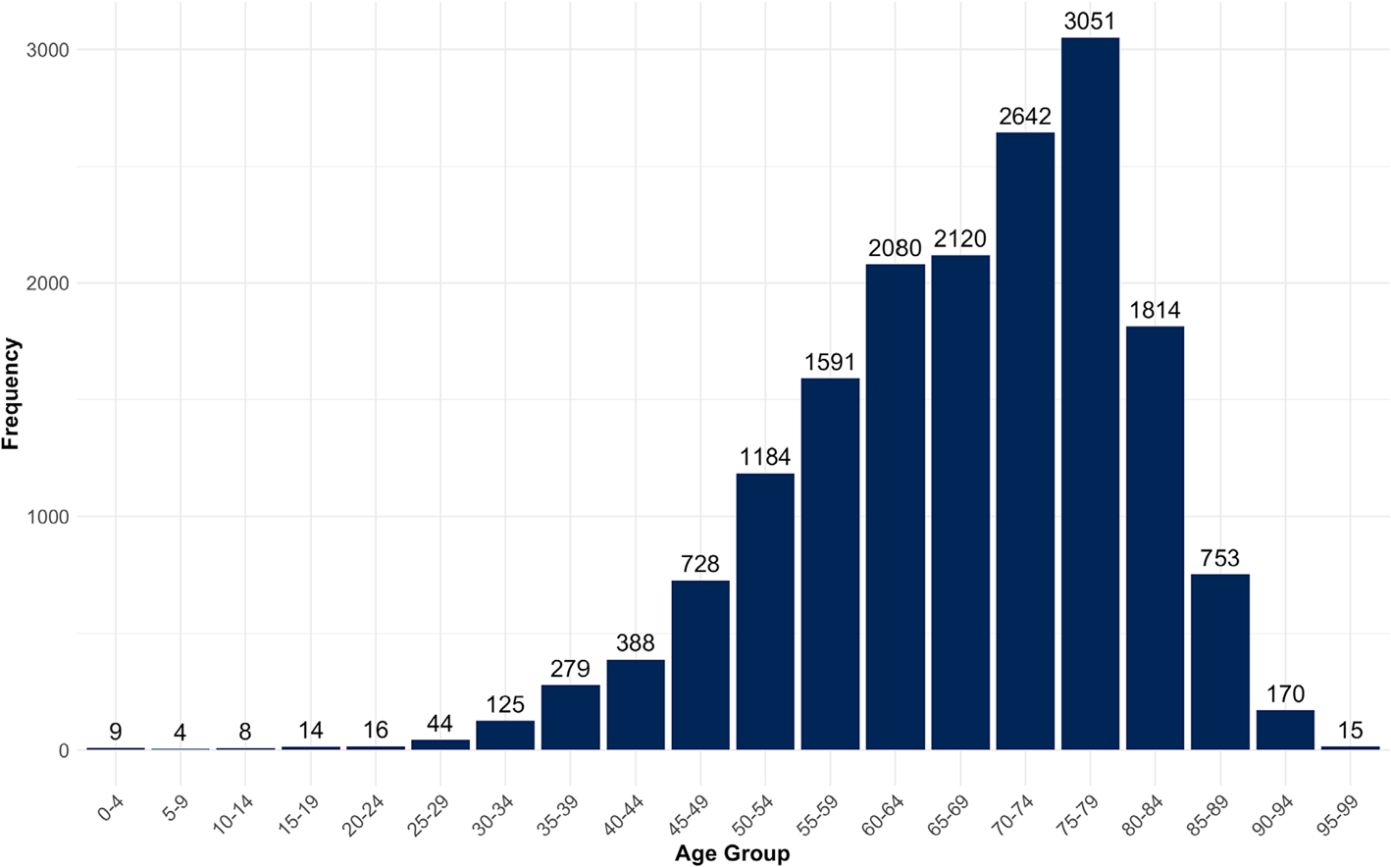
Age distribution of the study population at Chemnitz Hospital (Germany), 2018–2023

**Fig. 3:**
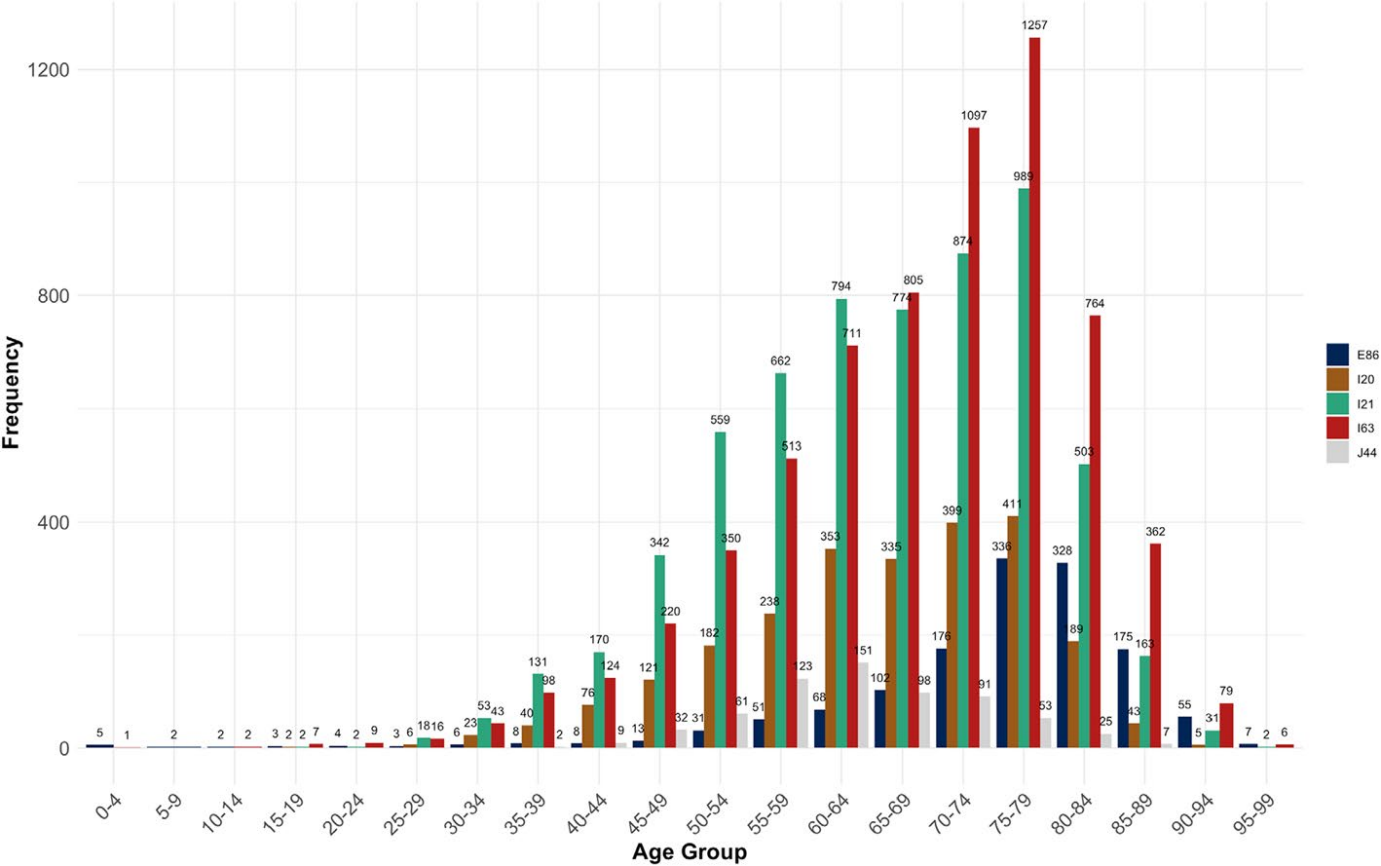
Age distribution of the individual diagnoses of the study population at Chemnitz Hospital (Germany), 2018–2023

The vast majority of patients was older than 50 years, with hardly no underaged treatment cases (Figs. 2 and 3). This can also be observed in the breakdown of the age distribution according to gender, with men (60.3%) generally overrepresented compared to women (39.7%) (Appendix 2).

### Description of climatic factors

**Fig. 4:**
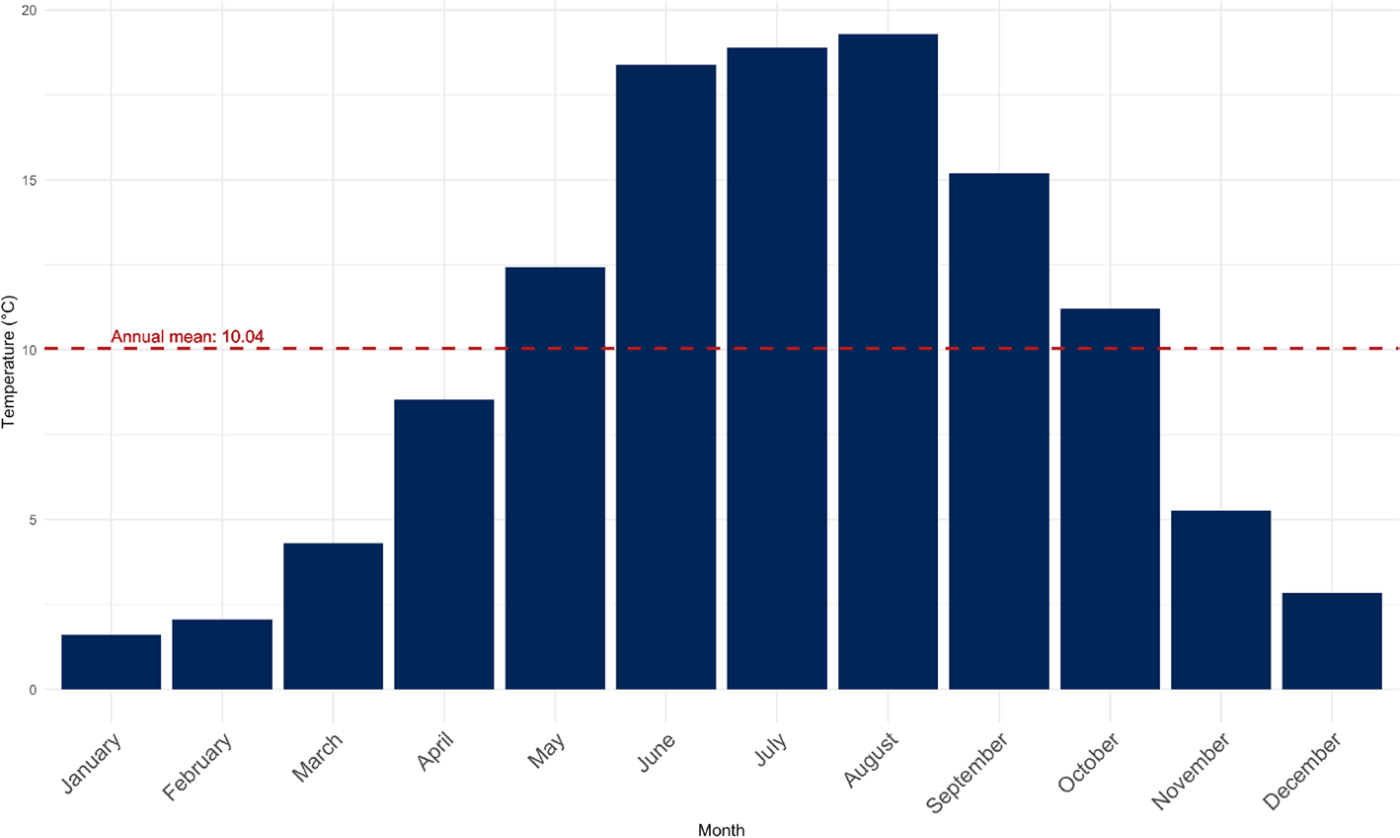
Monthly average temperatures in the observation period in Chemnitz (Germany), 2018–2023

During the observation period, the average temperature was 10.04°C. The months of May to October were above average, while the months of September to April below average (Fig. 4). A total of 427 warm days (≥ 23°C) were recorded, with 2021 (52 days) recording the fewest and 2018 (95 days) the most warm days. The number of heat days (≥ 30°C) was also highest in 2018 (15 days), while the lowest number of heat days was recorded in 2021 (1 day). A total of 59 heat days were recorded over the entire observation period (Table 3).

**Table 3:** Number of warm and heat days, and mean temperature with standard deviation (SD) per year in Chemnitz (Germany), 2018–2023

Year	Warm days ≥ 23°C	Heat days ≥ 30°C	Mean temperature (SD)
2018	95	15	10.36 (8.61)
2019	70	14	10.30 (7.55)
2020	64	7	10.28 (6.80)
2021	52	1	8.69 (7.68)
2022	71	12	10.25 (7.59)
2023	75	10	10.38 (6.62)
Total	427	59	10.04 (7.67)

### Correlation analysis

**Fig. 5:**
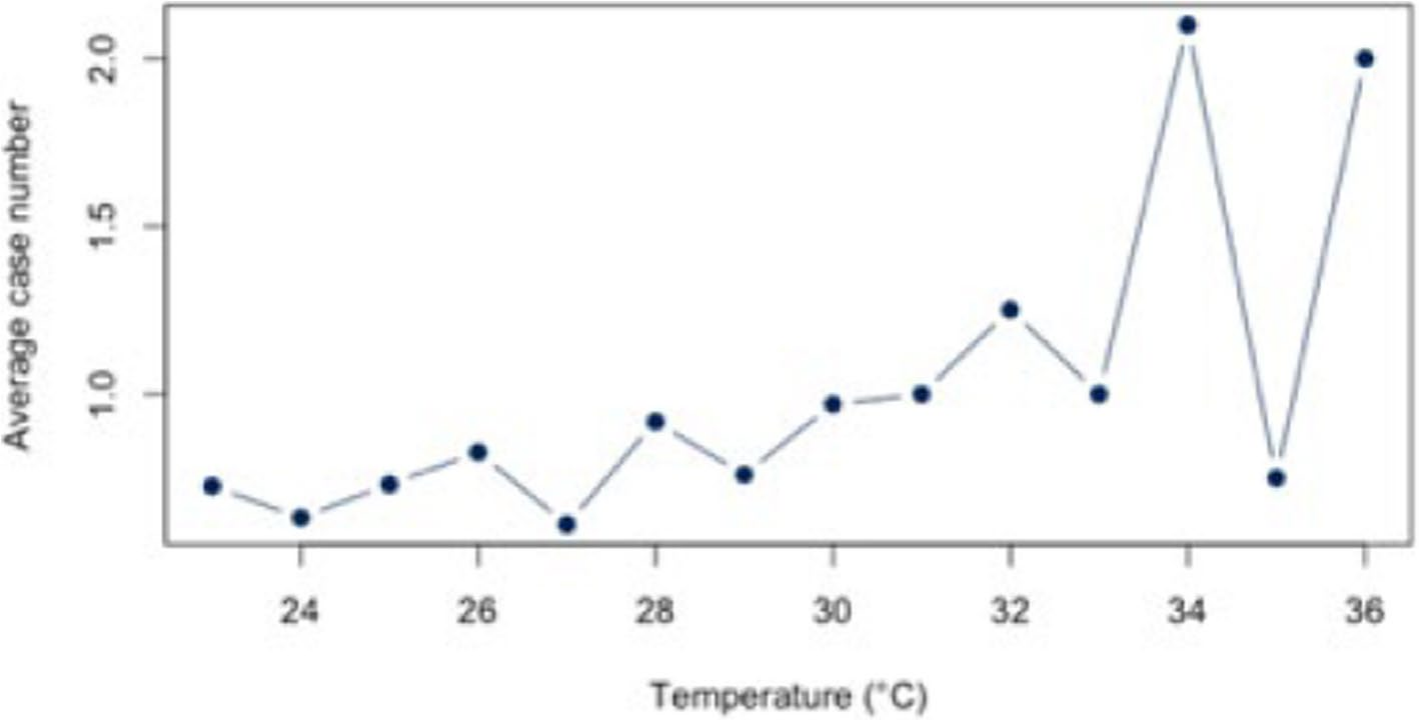
Hospital admissions due to E86 depending on maximum air temperature of ≥ 23°C at Chemnitz Hospital (Germany), 2018–2023

**Fig. 6:**
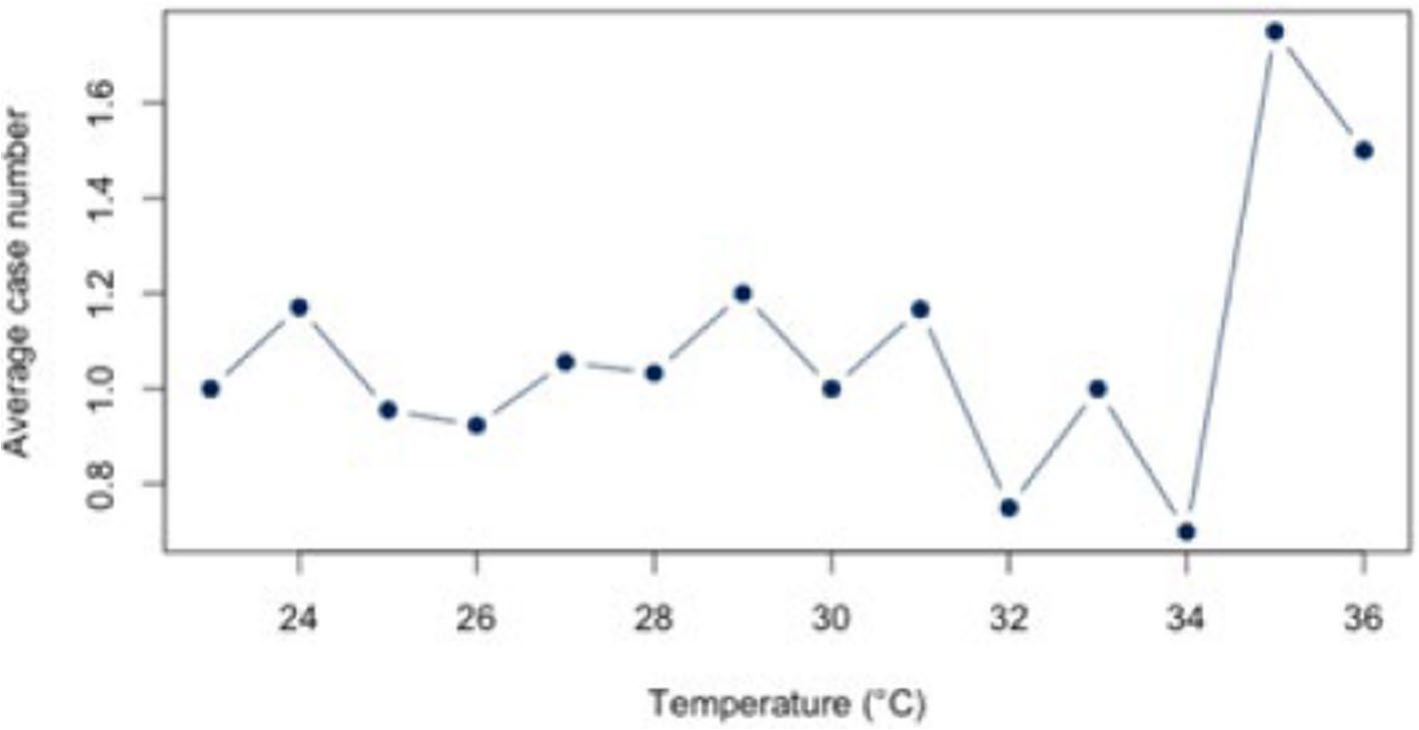
Hospital admissions due to I20 depending on maximum air temperature of ≥ 23°C at Chemnitz Hospital (Germany), 2018–2023

**Fig. 7:**
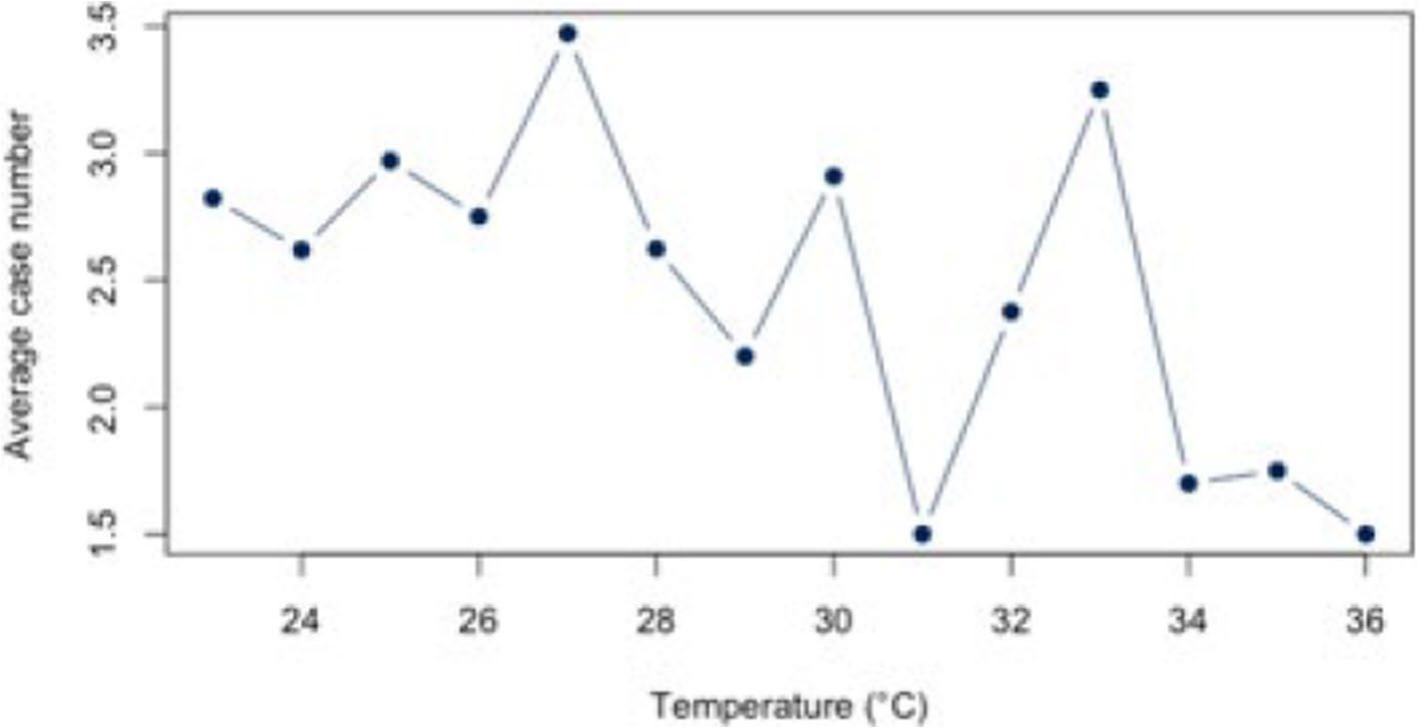
Hospital admissions due to I21 depending on maximum air temperature of ≥ 23°C at Chemnitz Hospital (Germany), 2018–2023

**Fig. 8:**
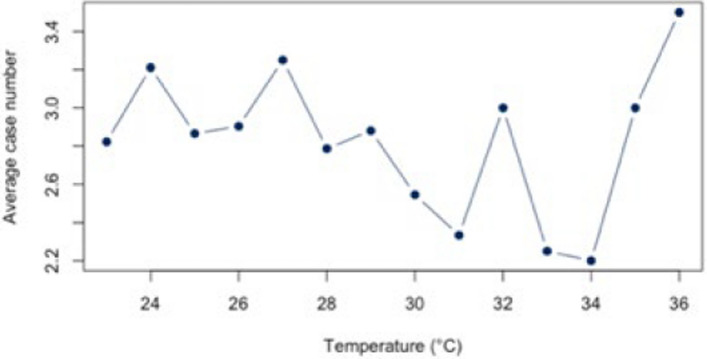
Hospital admissions due to I63 depending on maximum air temperature of ≥ 23°C at Chemnitz Hospital (Germany), 2018–2023

**Fig. 9:**
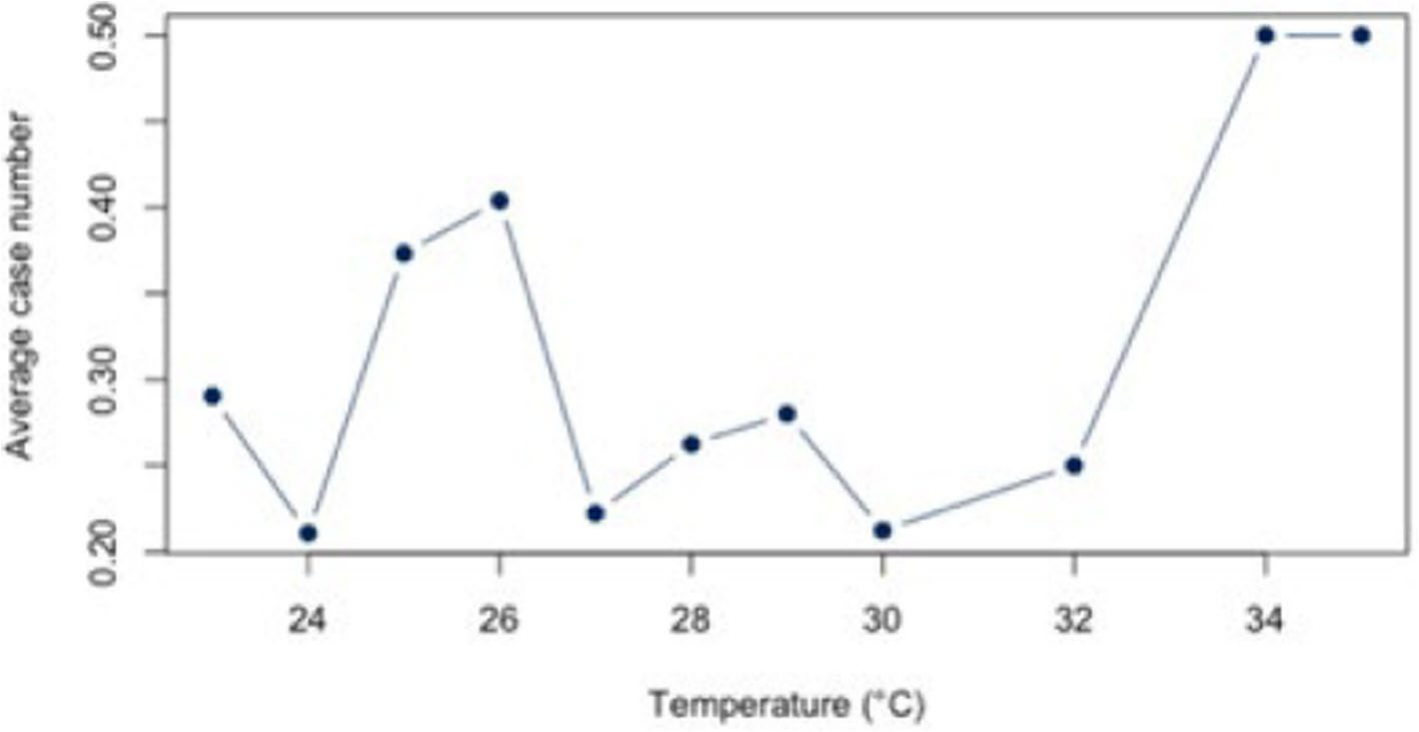
Hospital admissions due to J44 depending on maximum air temperature of ≥ 23°C at Chemnitz Hospital (Germany), 2018–2023

First, the correlation between heat and hospitalization for warm days with a threshold of ≥ 23°C was analyzed both for the individual diagnoses (Figs. 5, 6, 7, 8, and 9) and for all diagnoses combined (Appendix 3). For all diagnoses combined, this resulted in a correlation of 0.44 (95% CI = [−0.12;0.79]). The BF of 1.32 indicates inconclusive evidence. The analysis of the individual diseases revealed strong correlations for the diagnosis E86 (*r* = 0.69; CI = [0.25;0.89]) and I21 (*r* = −0.61; CI = [−0.86; −0.12]). The BF indicate a strong correlation between heat and hospitalization due to E86 (BF = 8.05) and I21 (BF = 3.93). Low correlations were found for the other diagnoses (Table 4).

**Table 4:** Results of the statistical correlation (r) analysis with p-Value (p), 95% confidence interval (CI) and Bayes Factor (BF) at Chemnitz Hospital (Germany), 2018–2023 (* *p* < 0.05, ** *p* < 0.01, *** *p* < 0.001)

Diagnosis code	≥ 23°C	BF	≥ 30°C	BF
All Diagnosis	*r* = *0.44 (p* = *0.114; 95% CI [−0.12;0.79])*	1.32	*r* = *0.88 (p* < 0.008**; 95% CI [0.39;0.98])	4.41
E86	*r* = *0.69 (p* = 0.006**; 95% CI [0.25;0.89])	8.05	*r* = *0.50 (p* = *0.258; 95% CI [−0.41;0.91])*	0.99
I20	*r* = *0.33 (p* = *0.250; 95% CI [−0.24;0.73])*	0.85	*r* = *0.53 (p* = *0.229; 95% CI [−0.38;0.92])*	1.05
I21	*r* = *−0.61 (p* = 0.020*; 95% CI [−0.86;−0.12])	3.93	*r* = -*0.48 (p* = *0.279; 95% CI [−0.91;0.43])*	0.96
I63	*r* = *−0.17 (p* = *0.564; 95% CI [−0.64;0.40])*	0.58	*r* = *0.54 (p* = *0.214; 95% CI [−0.36;0.92])*	1.07
J44	*r* = *0.50 (p* = *0.117; 95% CI [−0.14;0.85])*	1.35	*r* = *0.94 (p* = *0.057; 95% CI [−0.19;0.99])*	1.58

**Fig. 10:**
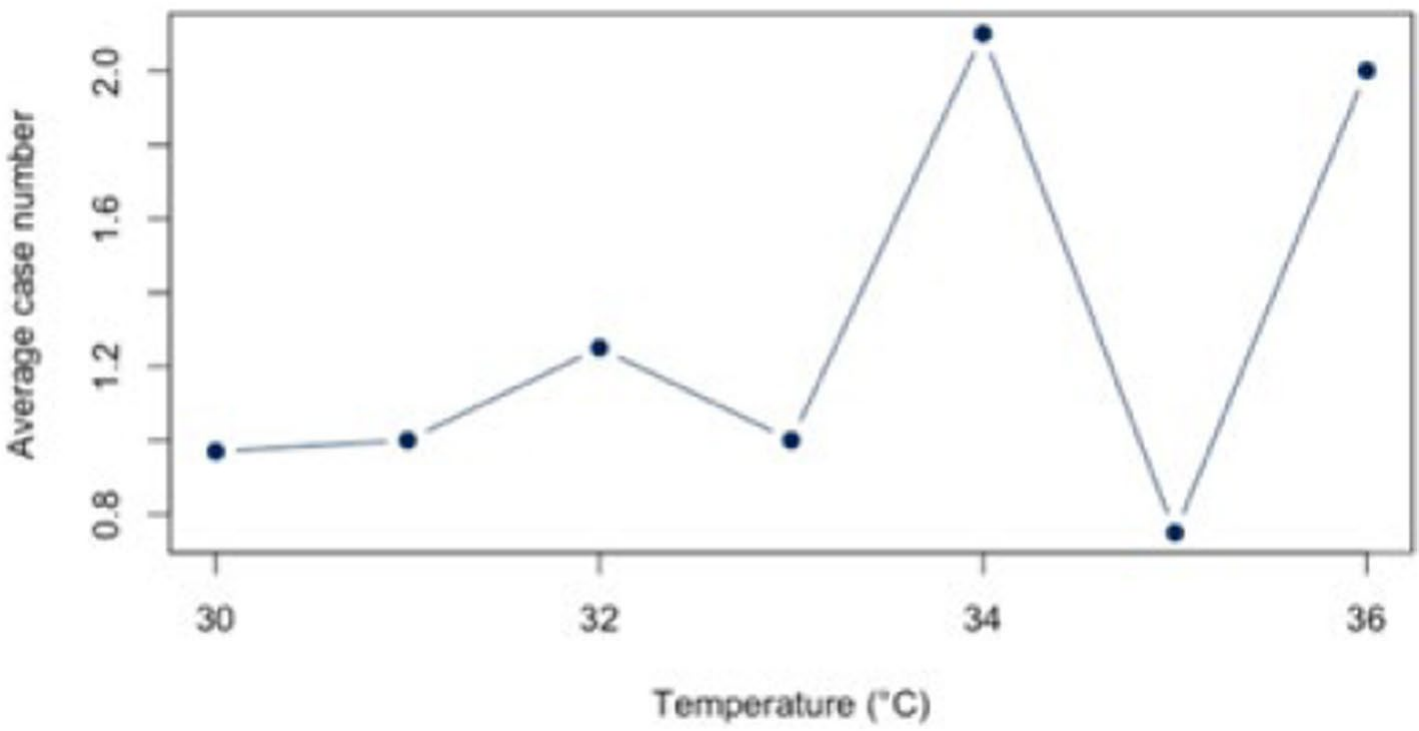
Hospital admissions due to E86 depending on maximum air temperature of ≥ 30°C at Chemnitz Hospital (Germany), 2018–2023

**Fig. 11:**
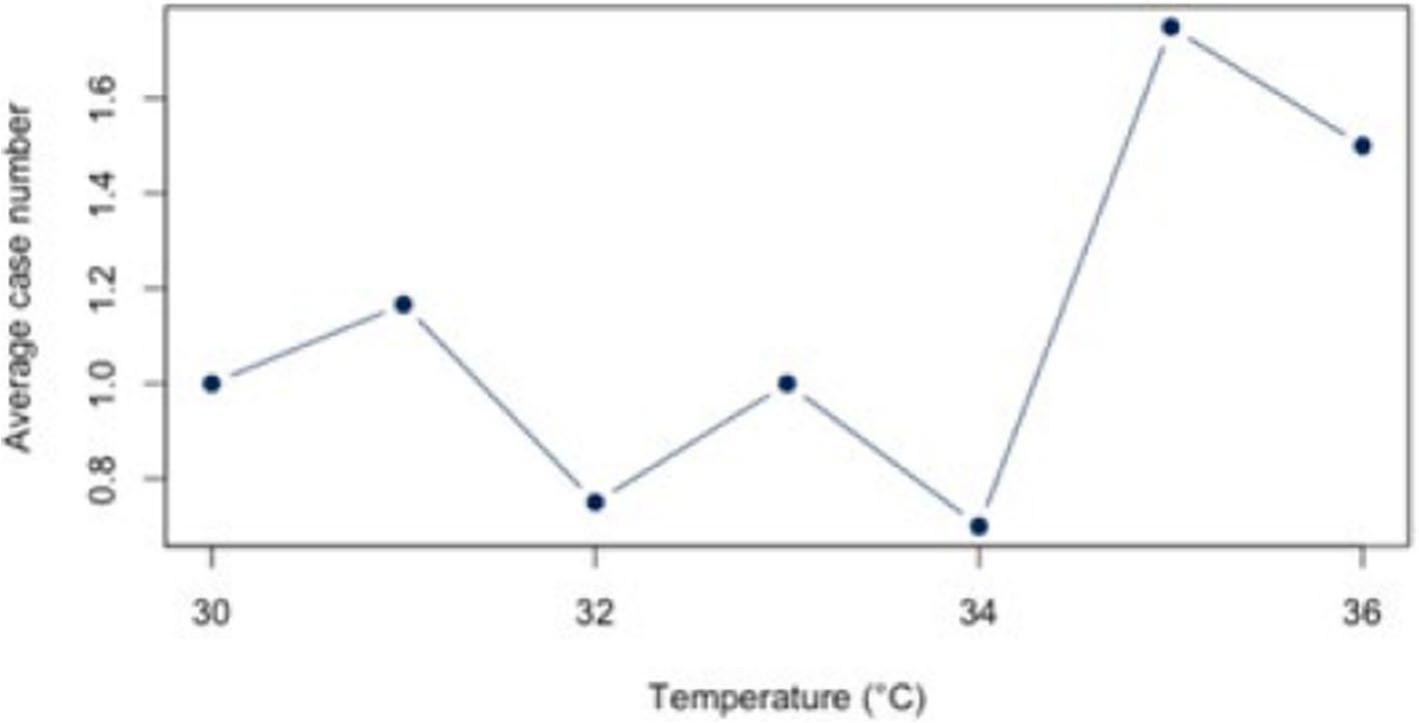
Hospital admissions due to I20 depending on maximum air temperature of ≥ 30°C at Chemnitz Hospital (Germany), 2018–2023

**Fig. 12:**
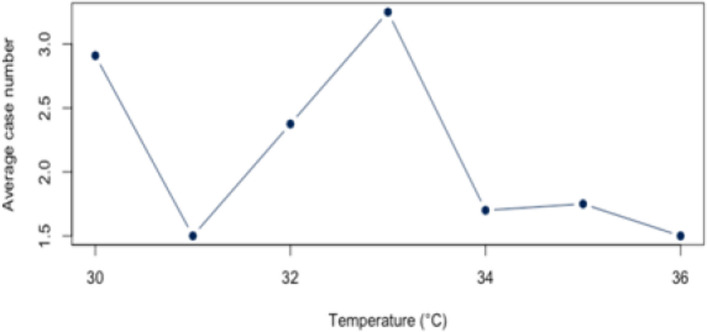
Hospital admissions due to I21 depending on maximum air temperature of ≥ 30°C at Chemnitz Hospital (Germany), 2018–2023

**Fig. 13:**
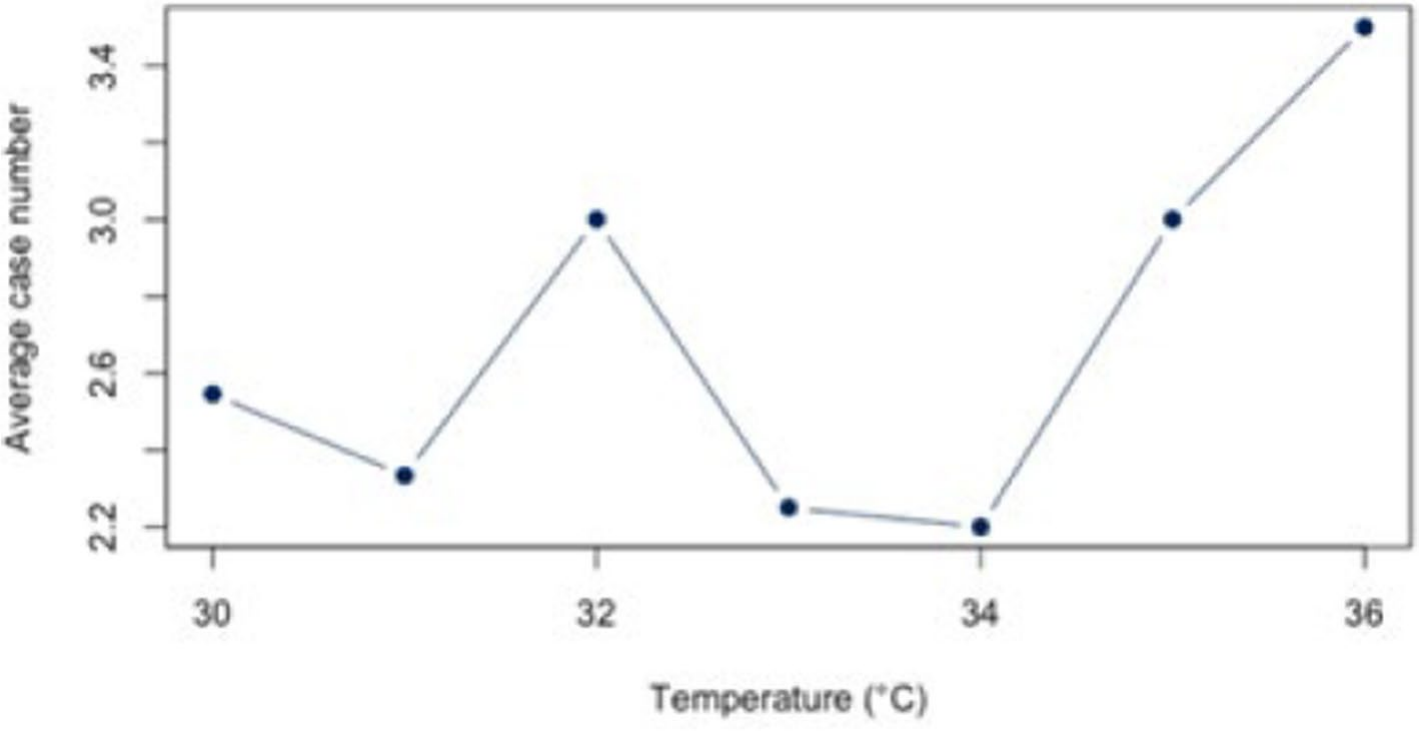
Hospital admissions due to I63 depending on maximum air temperature of ≥ 30°C at Chemnitz Hospital (Germany), 2018–2023

**Fig. 14:**
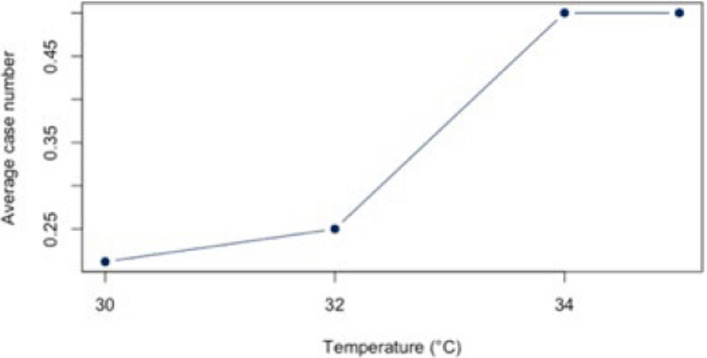
Hospital admissions due to J44 depending on maximum air temperature of ≥ 30°C at Chemnitz Hospital (Germany), 2018–2023

The analysis for the threshold of ≥ 30°C for all diagnoses combined (Appendix 4) showed a statistically significant correlation (*r* = 0.88, *p* < 0.01; CI = [0.387;0.983]) for all diagnoses combined. The BF of 4.41 also indicates a positive correlation between heat and hospital use. For the individual diagnoses (figs. 10, 11, 12, 13 and 14), the strongest correlation was found for the diagnosis J44 (r = 0.94, CI = [−0.19;0.99]). The BF of 1.48 indicates a lack of sensitivity of the data. A negative effect correlation (r = −0.48, CI = [−0.91;0.43]) was found for the diagnosis I21. The BF, which is shown in detail in Table 4, also indicates inconclusive evidence, as with most diagnoses.

### Linear regression analysis

**Fig. 15:**
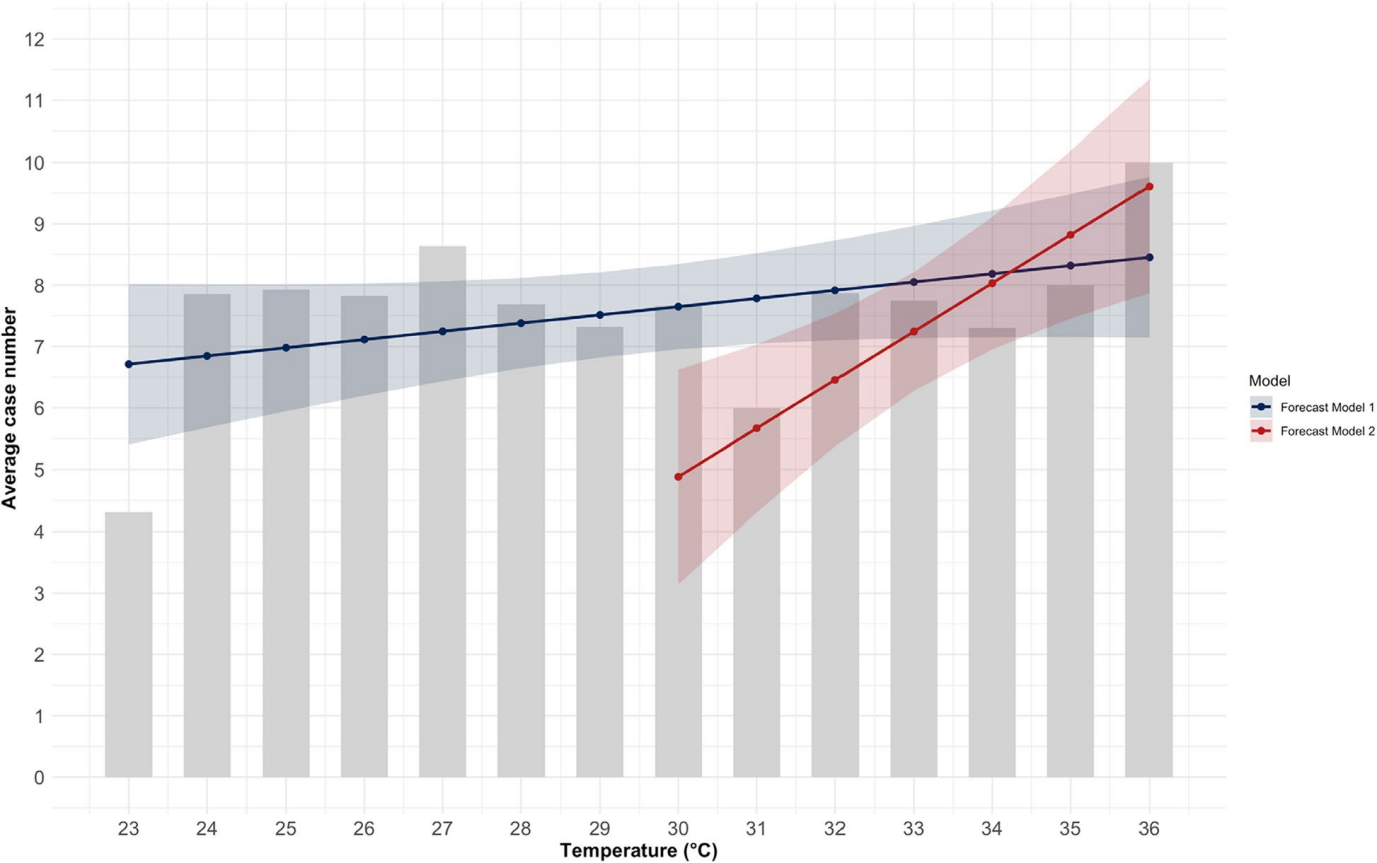
Observed patient admissions (mean) and fitted univariate linear regression lines of model 1 (≥ 23°C) and model 2 (≥ 30°C) at Chemnitz Hospital (Germany), 2018–2023

As a result of the univariate linear relationships identified, two linear models were developed using regression analysis (Fig. 15), which predict the number of patients as a function of temperature (Appendix 5). The first model referred to the threshold value of 23°C and enables predictions for warm days. This model (Table 5) showed an increase of 0.13 patient admissions for every 1°C increase in temperature, although the effect is not statistically significant (*p* = 0.11). The model for warm days was able to explain 12.8% of the variance in hospitalizations.

**Table 5:** Summary of the univariate linear regression analysis at Chemnitz Hospital (Germany), 2018–2023 (* *p* < 0.05, ** *p* < 0.01, *** *p* < 0.001)

Variable	Estimate (β) Model 1 (≥ 23°C)	Estimate (β) Model 2 (≥ 30°C)
Intercept	*3.634 (p* = *0.146)*	*−18.756 (p* = 0.029*)
Temperature	*0.134 (p* = *0.114)*	*0.788 (p* = 0.009**)
R-squared	0.195	0.779
Adjusted R-squared	0.128	0.735

The second model for the threshold value of 30°C showed an increase of 0.79 patient admissions for every 1°C increase in temperature, which was statistically significant (*p* < 0.01). The model for heat days was able to explain 73.5% of the variance in hospitalizations on heat days.

In addition, a multiple linear regression analysis was performed for both thresholds to adjust the influence of temperature for the impact of humidity, hours of sunshine, and air pressure. In multiple linear regression models adjusted for air humidity, sunshine hours, and air pressure, daily maximum temperature did not predict hospital admissions, neither in the model for warm days, nor in the model for heat days (Table 6).

**Table 6:** Summary of the multiple linear regression analysis at Chemnitz Hospital (Germany), 2018–2023 (* *p* < 0.05, ** *p* < 0.01, *** *p* < 0.001)

Variable	Estimate (β) Adjusted Model 1 (≥ 23°C)	Estimate (β) Adjusted Model 2 (≥ 30°C)
Intercept	*426.252 (p* = *0.248)*	*341.044 (p* = *0.358)*
Temperature	*0.009 (p* = *0.261)*	*0.137 (p* = *0.803)*
Air humidity	*0.058 (p* = *0.133)*	*0.060 (p* = *0.730)*
Sunshine hours	*0.507 (p* = *0.545)*	*0.456 (p* = *0.483)*
Air pressure	*−0.442 (p* = *0.360)*	*−0.440 (p* = *0.354)*
R-squared	0.384	0.752
Adjusted R-squared	0.110	0.256
F-statistic	0.309	0.434

### Non-linear regression analysis

**Fig. 16:**
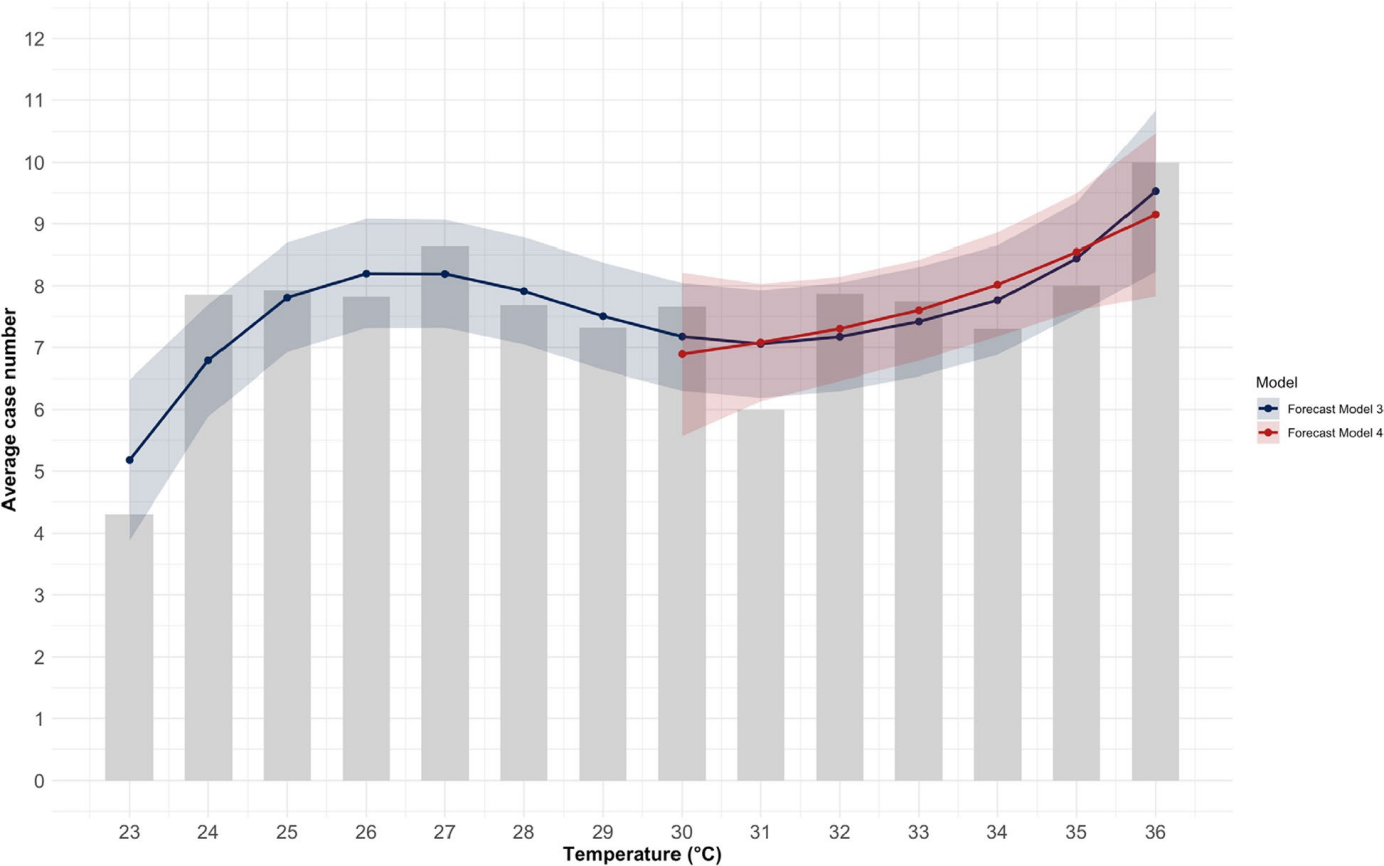
Observed patient admissions (mean) and fitted univariate non-linear regression lines of model 3 (≥ 23°C) and model 4 (≥ 30°C) at Chemnitz Hospital (Germany), 2018–2023

Two models were developed for the non-linear regression analysis (Table 7). Model 3 (warm days) showed a statistically significant association between temperature and hospital admissions (*p* = 0.035), with an explained variance of 63.4%. The effective degree of freedom (edf = 4.616) indicated a non-linear relationship, which was also reflected in the graphical representation (blue line in Fig. 16). The curve rises between 23°C and 26°C, then flattens out until 31°C, and then rises again. In contrast, Model 4 (heat days) showed no statistically significant association (*p* = 0.139), with an explained variance of 43.1%. The corresponding graphical representation (red line in Fig. 16) and the effective degree of freedom (edf = 1.411) suggested a rather linear trend.

**Table 7:** Summary of the univariate non-linear regression analysis with effective degrees of freedom (edf) at Chemnitz Hospital (Germany), 2018–2023 (* *p* < 0.05, ** *p* < 0.01, *** *p* < 0.001)

Variable	Estimate (β) Model 3 (≥ 23°C)	Estimate (β) Model 4 (≥ 30°C)
Intercept	*7.582 (p* < 0.001***)	*7.799 (p* < 0.001***)
Temperature	*edf* = *5.654; F* = *4.166; p* = 0.035*	*edf* = *1.411; F* = *2.461; p* = *0.139*
R-squared	0.634	0.431
Deviance explained	76.4%	56.5%

In model 3 (warm days) that was adjusted for air humidity, sunshine hours, and air pressure, a non-linear relationship was found for temperature (edf = 3,412). The other influencing factors had an edf of 1,000. None of the factors examined were statistically significant. The explained variance was 50.6% (deviance explained: 75%). In model 4 (heat days), linear relationships were found for temperature and air pressure (edf = 1.000) and non-linear relationships for humidity (edf = 2.151) and hours of sunshine (edf = 1.844). Here, too, none of the factors examined were statistically significant (Table 8).

**Table 8:** Summary of the multivariate non-linear regression analysis with effective degrees of freedom (edf) at Chemnitz Hospital (Germany), 2018–2023 (* *p* < 0.05, ** *p* < 0.01, *** *p* < 0.001)

Variable	Estimate (β) Adjusted Model 3 (≥ 23°C)	Estimate (β) Adjusted Model 4 (≥ 30°C)
Intercept	*238.29 (p* < 0.001***)	*7.80 (p* = *0.99)*
Temperature	*edf* = *3.412; F* = *1.601; p* = *0.241*	*edf* = *1.000; F* = *6.527; p* = *0.238*
Air humidity	*edf* = *1.000; F* = *0.209; p* = *0.662*	*edf* = *2.151; F* = *4.138; p* = *0.308*
Sunshine hours	*edf* = *1.000; F* = *0.462; p* = *0.518*	*edf* = *1.844; F* = *2.650; p* = *0.375*
Air pressure	*edf* = *1.000; F* = *0.714; p* = *0.426*	*edf* = *1.000; F* = *11.169; p* = *0.185*
R-squared	*0.506*	*1*
Deviance explained	*75%*	*100%*

## Discussion

### Main findings

The objective of this study, to analyze and predict the relationship between heat exposure and hospital admissions using data from Chemnitz Hospital, was successfully achieved. The findings indicate that rising temperatures are associated with an overall increase in the utilization of inpatient healthcare services. This is supported by positive correlations observed on both warm and heat days, with stronger associations found on heat days. These observations are further substantiated by higher BF on heat days compared to warm days. This could also be due to the fact that on warm days there is a more curvilinear relationship. The regression analyses indicate that the relationships in the range from 23°C to 29°C are non-linear and become more linear at 30°C and above. In addition, the adjustment for humidity, air pressure, and sunshine hours also shows an influence of these variables. It should be noted that no significant effects can be detected after the adjustments.

The analysis of specific diagnoses revealed that the increased hospital burden is primarily driven by rising case numbers for volume depletion (E86), COPD (J44), and angina pectoris (I20). The diagnosis myocardial infarction (I21) shows an overall negative correlation with temperature, with the effect being more pronounced on warm days than on heat days. While most results were not statistically significant, the BF suggest that the available data may lack sufficient sensitivity to reliably distinguish between the null and alternative hypotheses. Thus, it remains unclear whether no association exists or whether the statistical power was insufficient.

Four models were developed in this study, two linear and two non-linear, with thresholds starting at 23°C for warm days and 30°C for heat days. In addition, each model was adjusted for humidity, sunshine hours, and air pressure.

Linear analysis showed an increase in patient admissions on warm and heat days, but this was only statistically significant on heat days. The adjustment showed a negative relationship for both thresholds for air pressure and a positive relationship for humidity and sunshine hours. However, no statistical significance could be determined in either case. The results should therefore be interpreted with caution. It is noticeable that the explained variance in linear regression was always higher for heat days than for warm days. This can possibly be explained by the fact that relationships are non-linear on warm days. This is supported by the non-linear analyses.

The non-linear analyses indicate, that on warm days, a non-linear relationship with a change in monotony prevails. Accordingly, the explained variance in the non-linear univariate models for warm days was significantly higher than in the linear models. For heat days, on the other hand, the explained variance in the univariate non-linear model fell compared to the linear regression, which indicates a weaker fit compared to the linear analysis. A statistically significant influence of temperature can be observed in the non-linear model for warm days, whereas no statistical significance was found for heat days. Adjusting for the effects of air pressure, humidity, and hours of sunshine shows no significant effect for either threshold value. Also, the explained variance of 100% on heat days indicates overfitting. These are known problems with models of this type [31, 32] and limit a meaningful interpretation of the results of individual influencing factors. The interpretation of the results of the non-linear models for heat days should therefore be carried out with particular caution. In the future, other statistical analysis methods such as the SARIMA (Seasonal Autoregressive Integrated Moving Average) model should be used to investigate these seasonal interaction effects.

### Scientific context

In general, the findings of this study correspond with the results found in the literature [4, 28] and expand on them with regional findings for Saxony. Established methods were used that were also applied in other similar studies in Germany, which examined both linear and non-linear relationships in heat-related morbidity [33]. In addition, the results have once again shown that general linear models can be an appropriate methodological approach for predicting hospitalizations and can be at least equivalent to more complex approaches, as was demonstrated in a previous comparison [34].

However, the results of the individual diagnostic analyses do not fully correspond to scientific findings and require more detailed discussion. The analyses of diagnoses E86, I20, and J44 showed a positive correlation with heat. These results are consistent with our previous study [5–7], supporting the notion that volume depletion, angina pectoris, and COPD are particularly susceptible to rising temperatures [5–7].

The results for the diagnoses I21 and I63 are unexpected and surprising. For I63, a negative correlation was observed at the threshold of ≥ 23°C and a positive correlation at ≥ 30°C. To our knowledge, this shift in effects has not been identified in other studies and is therefore difficult to contextualize within the literature. However, it should be noted that these results are not statistically significant and it is not possible to make a clear conclusion from these results. This is further complicated by the fact that the cardiovascular disease I21 showed a general reduction in cases with increasing temperature in our dataset. While reductive effects in cardiovascular diseases due to heat can occasionally be observed in the literature, such as in the meta-analysis by Xu et al. [4], where one out of five included papers reported this, it remains an exception. Similar findings can also be observed in mortality studies. For instance, a study by Ragettli et al. reported overall increased mortality during heatwaves but identified reduced mortality in the circulatory systems category [35].

It can be concluded that the analyses fundamentally align with current literature, correspond to the scientific consensus, and provide a valuable contribution to understanding regional hospital occupancy during heat events in Germany. However, it should be noted that the increased utilization of inpatient healthcare services in Germany during the summer months may also be related to school holidays and, for example, the closure of outpatient providers [36]. Integrating outpatient data would therefore be highly valuable, especially since a study by Wen et al. [37] reported a higher odds ratio for heat-related conditions in the outpatient sector compared to the inpatient sector in Thailand. The absence of outpatient data in the present analysis may therefore lead to an underestimation of the overall effect of heat on healthcare utilization.

### Strengths and weaknesses

To our knowledge, this is one of the first times in Germany that it has been possible to predict hospital utilization in relation to heat. A key strength of this study lies in the use of an extensive dataset comprising over 15,000 patients and more than 17,000 treatment cases within a six-year period. Compared to other studies in Germany, which used smaller sample sizes [5–7] or less region-specific data [21], this study was able to examine a larger number of diagnoses individually, yielding more detailed results. Additionally, we also have a larger sample size than in previous analyses. Furthermore, various linear and non-linear effects were investigated, and other climatic factors besides maximum temperature were also controlled for. However, other patient-specific factors (e.g., age) that may also be relevant according to the literature were not taken into account [38]. The available data did not allow meaningful analysis of risk factors such as patient age and gender, which is why no model adjustments were made for these variables, even though they may be relevant according to the literature [5–7]. Data quality and availability also limited the analyses, as both climate and treatment data are secondary data not originally collected for research purposes. It should also be noted that the calculations are based on aggregated data, which significantly limits the statistical significance and, in particular, the generalizability of the results. The use of aggregated data might have limited statistical power, but was also done to deal with autocorrelation. Among other things, it is questionable what influence the COVID-19 pandemic had on the data collected during the study period. Given the low case numbers in the single-digit range for predictions, it is in general questionable whether such models are meaningful for individual hospitals. Integrating additional indicators of healthcare utilization, such as outpatient services, may provide a more comprehensive understanding of the effects of acute heat on healthcare utilization. A limitation of this study is also, that not all possible diseases were included in the analysis. However, the selection of diseases was based on a thorough review of the literature, which reduces the risk of omitting relevant conditions and increases the likelihood that the most significant heat-related diseases are represented in the analysis. Furthermore, the climate data were obtained from only one weather station, which does not reflect intra-urban variations in heat and its effects. In addition, the effects of patients from the surrounding area who do not live directly in Chemnitz but are treated there are not fully recorded. Furthermore, the study design is not suitable for uncovering possible causality. Future studies using longitudinal or case-crossover designs could help strengthen the evidence for potential causal associations.

### Practical implications and future steps

The findings of this study suggest a relationship between heat and healthcare system utilization. The investigations indicate that the correlations for warm days (≥ 23°C) follow a non-linear pattern and that a linear correlation follows from 30°C onwards. This highlights not only that heat is a relevant factor in the burden on the healthcare system, but also the importance of preventive measures—especially for moderate heat. Currently, the City of Chemnitz has primarily been monitoring the effects of heat exposure [39]. Although monitoring in accordance with the recommendations of the European Environment Agency (EEA) is highly relevant and forms the basis for further measures [40], targeted prevention measures should be implemented in the future. To this end, a heat action plan should be developed, based on the recommendations of the EAA and the WHO. This involves, in particular, early warning systems, targeted awareness campaigns, a focus on particularly vulnerable risk groups, and the adaptation of healthcare and urban planning to heat-related health risks [40, 41]. The findings of this study can serve as a supporting basis for the development of the aforementioned prevention approaches. Also, the models developed in this study can thus serve as a basis for the future development and implementation of prediction models. However, they should be further expanded in the future, e.g. by considering more diagnoses. Ideally, no aggregated data should be used, so that time series analyses can be performed instead. Alternatively, approaches could be pursued that enable predictions of resource utilization for specific regions, such as Saxony as a whole. One approach could involve aligning with the state government's plans for inpatient medical care in Saxony, which define the planning regions of North Saxony, East Saxony, and Southwest Saxony (Sächsisches [42]). During the COVID-19 pandemic, a tool named DISPENSE for hospital resource utilization, based on these three planning regions, was developed and applied [43]. Further research is needed to investigate the relationships within these planning regions to develop predictive models. This study provides important insights into the diseases examined and the methodological approach. Future studies should also consider additional factors such as age, gender or pre-existing conditions [5–7], that may also explain further variance in the model. Furthermore, the integration of data from outpatient care and further risk factors such as gender or age [5–7], and potentially other variables such es genetic variation [44], could be useful for optimizing the models.

In general, from a methodological perspective, it is recommended to validate the models, especially given the low daily case numbers. Further studies on the seasonal burden of inpatient healthcare in Germany should also be conducted to better understand potential applications of such models. Other approaches should be examined as well, in particular time series analyses.

## Conclusion

This study uses the example of Chemnitz Hospital to examine the relationship between heat and the use of inpatient health services. The results show that even moderate temperatures above 23 °C are associated with an increased hospitalization rate, and that morbidity rises even more sharply above 30 °C. While linear models are useful for heat days, non-linear approaches proved to be more explanatory, especially on warm days, but were sometimes prone to overfitting. Multivariate models did not provide consistently significant findings and should therefore be interpreted with caution and further developed.

The findings highlight the health impacts of rising temperatures and emphasize the need for preventive measures, such as early warning systems for heat waves, targeted information campaigns for risk groups, and the adaptation of health infrastructure. At the same time, the study underscores the urgency of comprehensive climate protection measures to limit the long-term health risks of climate change.

Future research should be based on expanded data sets, particularly outpatient data and other statistical methods based on raw data rather than aggregated data. In addition, further regional analyses should be conducted to better capture regional differences in the vulnerability and resilience of health systems and to develop more effective adaptation strategies. Therefore, similar analyses should be carried out in other regions and, based on these, target group-specific educational measures or the implementation of prediction models for patient admissions should be implemented.

## Supplementary Information

Supplementary Material 1.

## Data Availability

The data that support the findings of this study are held by the Data Integration Center of the Klinikum Chemnitz gGmbH. Due to privacy and data protection regulations, the data are not publicly available and can only be accessed upon request directly from the Klinikum Chemnitz gGmbH.
