# Supplementary material for: Analyzing and predicting patient admissions related to acute heat at the Chemnitz Hospital (Germany)
Source: Arch Public Health. 2025 Nov 26;83:292. doi: 10.1186/s13690-025-01789-9 (PMC12667145; doi:10.1186/s13690-025-01789-9)
Supplement: Supplementary file 1 — Supplementary Material 1. [file 13690_2025_1789_MOESM1_ESM.pdf]

Appendix

Appendix 1:

Model	Formula	Variable Description
Simple LM	$Y_i = \beta_0 + \beta_1 X_{i1} + \varepsilon_i$	Y_i: predicted number of patients on day i<br>X_{i1}: temperature<br>β_0: intercept<br>β_1: regression coefficient for temperature<br>ε_i: error term
Multiple LM	$Y_i = \beta_0 + \beta_1 X_{i1} + \beta_2 X_{i2} + \beta_3 X_{i3} + \beta_3 X_{i3} + \varepsilon_i$	Y_i: predicted number of patients on day i<br>X_{i1}: temperature<br>X_{i2}: relative humidity<br>X_{i3}: air pressure<br>X_{i4}: sunshine hours<br>β_0: intercept<br>β_j: regression coefficients<br>ε_i: error term
Simple GAM	$Y_i = \beta_0 + s_1(X_{i1}) + \varepsilon_i$	Y_i: predicted number of patients on day i<br>X_{i1}: temperature<br>s_1: smooth function for temperature<br>β_0: intercept<br>ε_i: error term
Multiple GAM	$Y_i = \beta_0 + s_1(X_{i1}) + s_2(X_{i2}) + s_3(X_{i3}) + s_4(X_{i4}) + \varepsilon_i$	Y_i: predicted number of patients on day i<br>X_{i1}: temperature<br>X_{i2}: relative humidity<br>X_{i3}: air pressure<br>X_{i4}: sunshine hours<br>s_j: smooth functions for each predictor<br>β_0: intercept<br>ε_i: error term

Appendix 2: Age and gender distribution

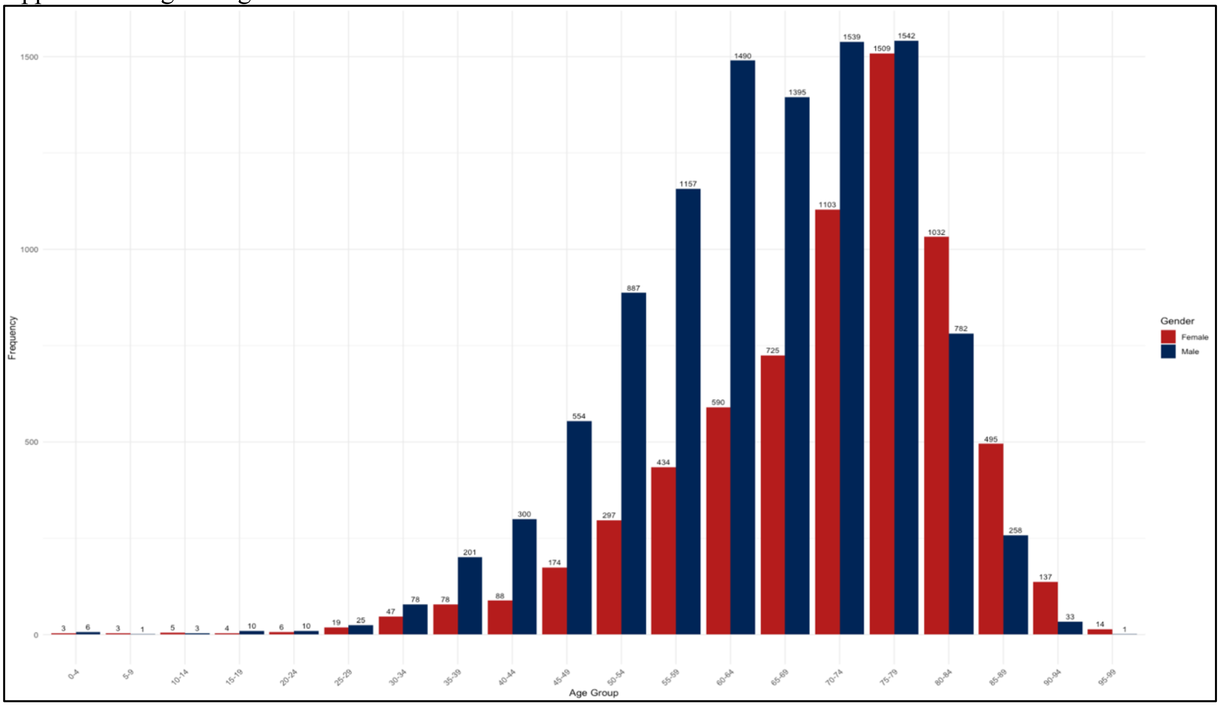

Appendix 3: Link between all diagnoses and temperature for ≥ 23°

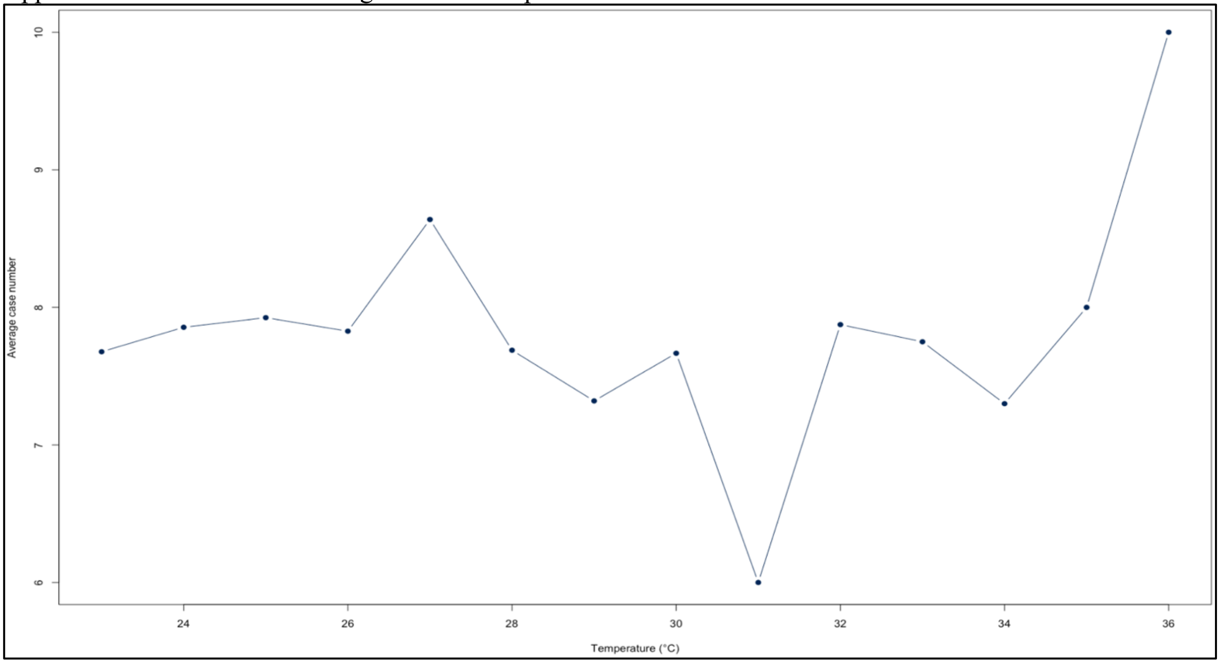

Appendix 4: Link between all diagnoses and temperature for ≥ 30°

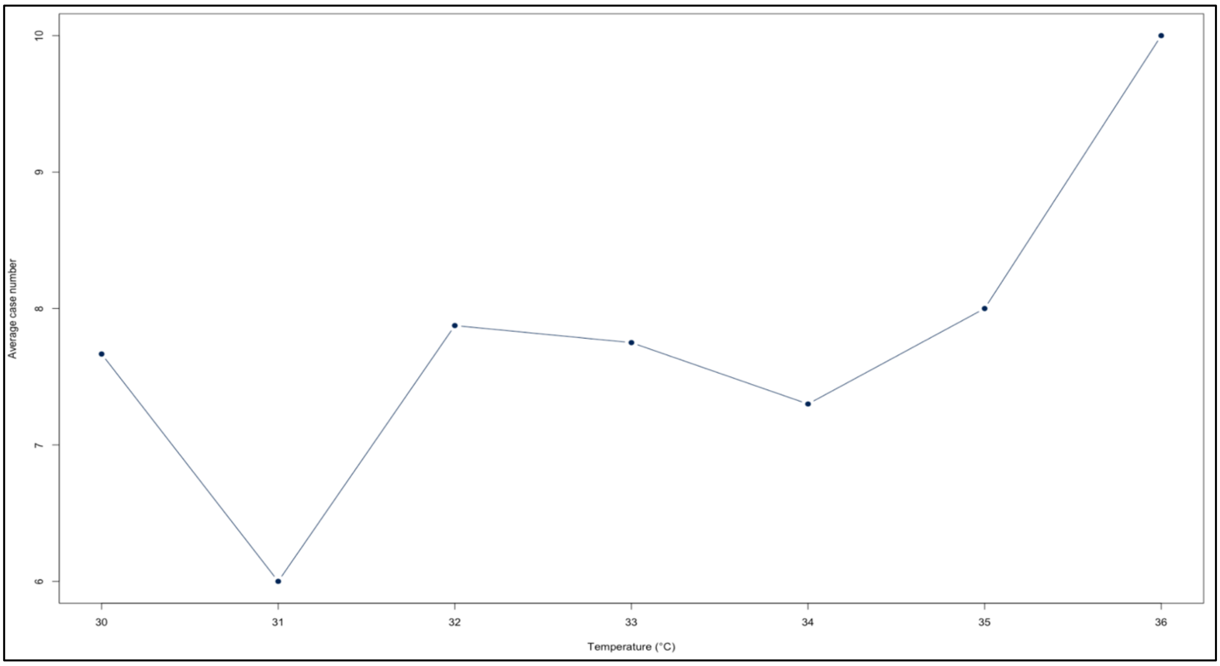

Appendix 5: Predictions of patient admissions

Temperature (°C)	Number of patient admissions – Model 1 (≥23°C)	Number of patient admissions – Model 2 (≥30°C)
23	6,71	Out of range
24	6,85	Out of range
25	6,98	Out of range
26	7,11	Out of range
27	7,25	Out of range
28	7,38	Out of range
29	7,52	Out of range
30	7,65	4,88
31	7,78	5,67
32	7,92	6,46
33	8,05	7,24
34	8,18	8,03
35	8,32	8,82
36	8,45	9,61
